# Vulnerability for Respiratory Infections in Asthma Patients: A Systematic Review

**DOI:** 10.7759/cureus.28839

**Published:** 2022-09-06

**Authors:** Sucheta Sharma, Nishat Tasnim, Kuchalambal Agadi, Ummul Asfeen, Jatin Kanda

**Affiliations:** 1 Internal Medicine, Medway Maritime Hospital, Gillingham, GBR; 2 Internal Medicine, Sacramento Veterans Affairs Medical Center, Mather, USA; 3 Division of Research and Academic Affairs, Larkin Community Hospital, Chicago, USA; 4 Internal Medicine, Saint Michael's Medical Center, Newark, USA; 5 General Medicine, Macclesfield District General Hospital, Macclesfield, GBR

**Keywords:** respiratory tract infection, immunosuppression, allergy and anaphylaxis, upper respiratory tract infections, respiratory viruses, asthma

## Abstract

Asthma is a non-communicable and long-term condition affecting children and adults. The air passages in the lungs become narrow due to inflammation and tightening of the muscles around the small airways. Symptoms of asthma are intermittent and include cough, wheeze, shortness of breath, and chest tightness. Asthma is very often underdiagnosed and under-treated in many regions, especially in developing countries. While many studies show that viral infections can precipitate asthmatic attacks, very few studies have been conducted to see if history or current asthmatic attack increases the risk of viral infections. Our study aims to determine the predisposition of asthmatics to develop various viral infections and susceptibility toward certain viruses that cause upper respiratory tract infections.

We performed a literature review of both published and unpublished articles. We included case reports, case series, reviews, clinical trials, cohort, and case-control studies, written only in English. Commentaries, letters to editors, and book chapters were excluded. Our initial search yielded 948 articles, of which 826 were rejected either because they were irrelevant or because they did not meet our inclusion criteria. We finally screened 122 abstracts and identified 24 relevant articles.

People with a history of asthma have an abnormal innate immune response, making them potentially slower in clearing the infection and susceptible to both infections and virus-induced cell cytotoxicity. Also, in these studies, deficiencies in the interferon alpha response of peripheral blood mononuclear cells and plasmacytoid dendritic cells have been observed in asthmatics, both adults and children. Asthmatics with a viral infection usually present with an acute exacerbation of asthma, represented by dyspnea and cough, with other prodromal symptoms including vomiting and general malaise.

The review includes an update on the relevance of dysregulated immune pathways in causing viral infections in asthmatic populations. It focuses on the evidence to suggest that people with asthma are at increased risk of viral infection, and viral infections in turn are known to precipitate and worsen the asthmatic status, making this a vicious cycle. The authors also suggest that further studies be undertaken to elucidate the pathophysiology and identify the critical therapeutic steps to break this vicious cycle and improve the quality of life for people with asthma.

## Introduction and background

Asthma is a heterogeneous disease, usually characterized by chronic airway inflammation. It is defined by the history of respiratory symptoms such as wheezing, shortness of breath, chest tightness, and cough that vary over time and in intensity, together with the variable expiratory flow limitation. Airflow limitations may later become persistent [1]. Both genetic predisposition and environmental factors play a role in the etiology of asthma. Recurrent asthmatic attacks are associated with chronic inflammatory changes in the lamina propria. As a result, there is a thickening of the basement membrane of the respiratory epithelium, hypertrophy of the respiratory smooth muscle layer, and an increased number of respiratory submucosal glands and epithelial goblet cells in the terminal bronchioles. The airway in asthmatics tends to be hypersensitive, developing frequent flare-ups to exposure to many irritants, such as allergens, cold, stress, and exercise. Inflammation resulting from this response induces mucous production, therefore, exacerbating the narrowing of the airway.

This inflammatory environment present in the airways of asthmatics could make them susceptible to airborne infections as pathogens find a friendly culture media in the swollen and narrow airways.

Though the exact mechanism of association between infections and asthma has not been established, with recent studies, there has been some progress in understanding the pathogenesis of increased incidence of respiratory infections in patients with asthma. Recent data provide overwhelming evidence of an increased rate of viral infection in patients suffering from asthma exacerbations requiring hospitalizations, with the most common prevalent virus being human rhinovirus (HRV). A high percentage of asthma exacerbation episodes reported among the pediatric population are attributed to viral infestations, mainly picornavirus, coronavirus, influenza, parainfluenza, respiratory syncytial virus (RSV), and rhinovirus [2,3]. Most viral infections are observed to have a grave impact on various aspects of asthma [4]. Almost half of the pediatric patients with asthma reported suffering from severe asthma exacerbations each year [5]. Factors like age and gender play a significant role in increasing susceptibility to viral infections in people with asthma [6,7].

The morphological changes in asthma increase the risk of morbidity and mortality by reducing the growth and function of the lung [8]. In 80-85% of children, viral respiratory infections are the crucial reason for asthma exacerbation [9]. However, adult patients infected with rhinovirus develop the most severe symptoms [8]. Among the wide range of symptoms, wheezing and cough are the most common. Other symptoms include shortness of breath, wet cough, nasal discharge, sore throat, headache, joint pain, muscle pain, fever, eye discharge, and vomiting [8]. Patients infected with rhinovirus have a three-fold increased risk of developing recurrent wheeze compared to other viruses [10]. The severity of these symptoms stands on a four-point scoring system: none, mild, moderate, and severe [11]. Mild symptoms include mild stifling or runny nose, which do not affect daily activities, moderate symptoms affect daily activities but not sleep, whereas sleep and breathing difficulties are part of severe symptoms. Symptoms of viral asthma exacerbation follow the criteria of mild, moderate, and severe [11]. Peak expiratory flow rate (PEF) is a critical biomarker of lung function, which is also affected by acute exacerbation. Moreover, the benchmark for loss of asthma control includes at least moderate asthma symptoms and either reduction in PEF of 20% or more, or the use of albuterol for more than two days per week [10]. However, Olenec et al. classified acute exacerbation into four categories as follows: no symptoms, solitary cold symptoms, solitary asthma symptoms, and combined cold and asthma symptoms [12].

Viruses were detected mostly either with solitary cold symptoms or combined cold and asthma symptoms. During solitary asthma symptoms, virus detection rates were similar to asymptomatic children [13]. Spring and fall are the peak seasons for asthma exacerbations [11]. During the fall season, most viruses are detected in September, termed the "September Epidemic" [9,11,13]. Causes of the "September Epidemic" in children encompass the return to school after a long break, exposure to viruses and airborne allergens, etc. [11]. These patients require hospitalization or an unplanned visit to the doctor. The prime risk factors comprise preceding acute exacerbation, allergy, poorly controlled asthma, young age, and viral respiratory infections [9]. Interconnection between viral infections and allergies elevates the risk of exacerbation to a greater level [14]. Therefore, further studies are needed to confirm if patients with a history of asthma are at increased risk of viral infections.

While there is enough evidence of viral infections precipitating asthma attacks, the predisposition of asthmatics to develop viral infections has not been clearly demonstrated. We reviewed the existing literature aiming to determine the vulnerability of asthmatic patients to contracting viral and other infections.

## Review

Methodology

**Figure 1:**
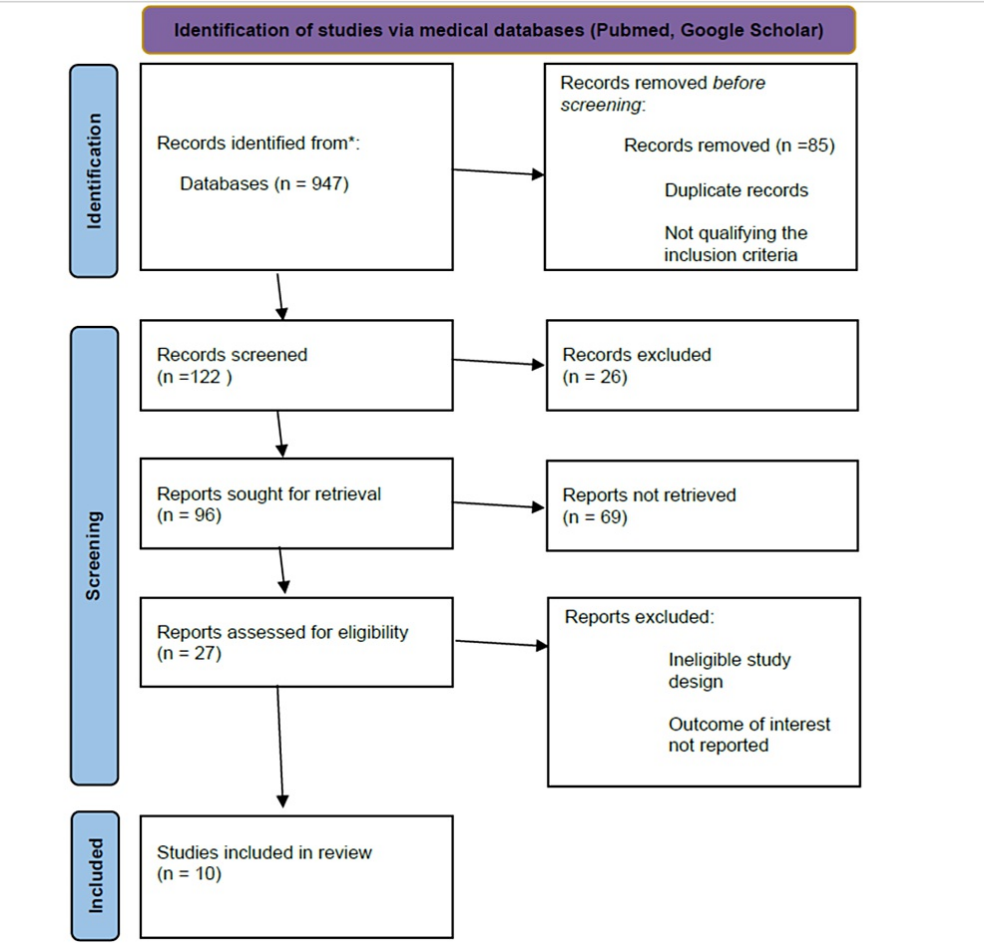
PRISMA flowchart showing the screening of studies PRISMA: Preferred Reporting Items for Systematic Reviews and Meta-Analyses.

Medical databases PubMed and Google Scholar were used for literature search through the following Medical Subject Heading (MESH) keywords: “viral infections,” “infections,” and “asthma.” We have included articles published from the year 2000 to 2021 in the English language only. We have included reviews, randomized controlled trials, and observational studies for data extraction and analysis while excluding unpublished articles, chapters from books, commentaries, and letters from editors. A total of 10 studies were selected after the review as they fit the selection criteria (Figure 1), making this a traditional literature review. Since it is a literature review, we did not perform a quality assessment of the selected articles. The majority of data obtained from the included studies were through the use of the reverse transcription polymerase chain reaction (PCR) on nasopharyngeal specimens from patients with asthma to detect and identify viral strains.

Results

The results of the studies reviewed demonstrated an increased vulnerability toward lower respiratory tract infections in asthmatic populations as compared to populations with normal lung physiology, following an experimental HRV inoculation [15,16]. There are multiple etiologies of asthma exacerbation including viruses, bacteria, allergens, irritants, and occupational exposures, of which respiratory viruses are the most frequent cause [17]. In an observational study using experimental rhinovirus inoculation, a clear difference was noted between pre and post-viral challenge measurements of exhaled NO2 and eosinophil density in the nasal fluid in asthmatics compared to the healthy controls [18]. The observations made from a similar study implied that homeokinesis of the respiratory system is altered in asthmatics, which affects its capacity to respond to external stimuli like HRV [18]. Moreover, during an analysis of 60 samples of nasopharyngeal aspirates collected from children below 17 years of age with acute asthma exacerbations in Iran, rhinovirus was reported to be positive in 20% of patients, RSV in 8%, adenovirus in 8%, and influenza virus in 1.6% [19]. A decrease in innate and adaptive immunity during childhood, especially in infancy, has been found to increase the susceptibility to viral respiratory infections, especially among those who are at increased risk of asthma [19]. A case-control study performed in asthmatic children aged two to 17 years in 2007 detected one or more virus-positive respiratory specimens in 63% of children with asthma during acute exacerbation and 23% of children with well-controlled asthma [20]. Other respiratory viruses detected in respiratory samples of asthmatic children include enterovirus, coronavirus, metapneumovirus, parainfluenza virus, and bocavirus [21,22]. The second most common cause of upper respiratory infections causing acute exacerbations of asthma following viruses are bacteria such as *Chlamydia pneumoniae*, *Mycoplasma pneumoniae*, *Streptococcus pneumoniae*, and *Haemophilus influenzae* [22,23]. A prospective cohort study conducted in 2013 on pregnant females reported an increased incidence of the common cold among asthmatic patients as compared to non-asthmatic patients [24]. In the same study, 31% of asthmatic pregnant women and 18.8% of pregnant women without asthma had at least an episode of PCR-positive cold during pregnancy [24]. The significant results from all the reviewed studies have been compiled below in Table 1.

**Table 1:** Characteristics and summary of main findings from the included studies COPD: chronic obstructive pulmonary disease; HRV: human rhinovirus; RSV: respiratory syncytial virus; RV16: rhinovirus 16.

Year of study	Author	Study design	Results	Type of study cohort
2012	Althani et al. [13]	Observational study	The most prevalent viruses affecting people with asthma are rhinovirus and coronavirus.	200 adult patients with COPD and asthma seeking care for respiratory infections.
2010	Olenec et al. [12]	Observational study	Respiratory viruses were detected in up to half of weekly samples obtained from school-aged children with asthma, and HRV infections were major contributors.	58 children (6-8 years) with asthma were enrolled and followed for 5 weeks.
2007	Khetsuriani et al. [11]	Observational study	Children with acute asthma exacerbations were sixfold more likely to be infected with respiratory viruses than children with controlled asthma.	65 children (2-17 years) with asthma exacerbations as cases, 77 children with well-controlled asthma as controls.
2019	Hassanzad et al. [19]	Observational study	Viruses detected from nasopharyngeal swabs of the asthmatic population (under 17 years of age) in order of prevalence are rhinovirus, RSV, adenovirus, and influenza.	60 patients (under 17 years) hospitalized with asthma exacerbations were recruited.
2020	Taka et al. [25]	Literature review	The presence of variable intracellular and extracellular expressions in people with asthma compared to people with no asthma, which can be altered by different viruses.	Not applicable
2013	Murphy et al. [16]	Prospective cohort study	Pregnant women with a history of asthma are more prone to flu than those with no history of asthma. Flu during pregnancy is associated with increased mental health issues and a higher likelihood of preeclampsia.	168 pregnant women with asthma as cases and 117 pregnant women without asthma as controls.
2019	Sinha et al. [18]	A prospective, observational, follow-up study	In asthma, loss of adaptive capacity and unstable physiological responses increases susceptibility to viral infections.	12 non-smoking individuals with asthma and not using steroids as cases and 12 non-smoking healthy individuals as controls.
2000	Grünberg et al. [26]	Randomized control trial	RV16 infection causes only minor worsening of airway inflammation. Inhaled corticosteroids do not improve or worsen the RV16-induced changes in airways.	203 patients (above 20 years) with chronic respiratory diseases were randomized to receive either laninamivir octanoate or oseltamivir.
2019	Verduyn et al. [20]	Observational study	Significantly lower IgG levels contributed to virus-induced asthma exacerbations.	82 patients hospitalized for asthma exacerbations over a year were recruited.
2019	Jha et al. [21]	Observational study	Female patients with asthma more often develop influenza than those without asthma. However, they require shorter hospital stays, less mechanical ventilation, and better outcomes.	133 adult asthmatic patients presenting with influenza-like symptoms from 3 different hospitals in the UK were recruited.

Discussion

Morphological and Immunological Changes in Asthma That Lead to Viral Infections

Asthma is a dynamic disease in which mechanical and inflammatory pathways interact in an exaggerated manner, resulting in unstable inflammatory responses to external triggers [18]. Certain immunological factors such as microRNAs (miRs), interferon (IFN)-α, IFN-β, and IFN-λ, forced expiratory volume in one second (FEV1), interleukin (IL)-11, nuclear factor-kappa B (NF-κB), and toll-like receptor 7 (TLR7) aid in the pathogenesis that leads to different viral Infections [21,22,25,27,28].

The deficit in both acquired and innate immune systems collectively lead to impairment of the physiological airway barrier facilitating the entry of the viruses into the respiratory tracts. miRs are small non-coding RNAs, classified as extracellular and intracellular based on their topographical identification, that function as dynamic post-transcriptional regulators of gene networks, playing a crucial role in the regulation of biological processes such as the proliferation of cells when exposed to antigens [25]. Modulation of miR expression has been used to describe key roles of miRs in epithelial function and emerging studies implicate specific miRs in controlling epithelial cell processes such as regulation of cellular differentiation, determination of epithelial cell fate (cell proliferation and death), initiation and regulation of antimicrobial immunity, fine control of inflammatory responses, and activation of intracellular signaling pathways [25]. Such control of epithelial cell functions is likely to be vital to fine-tuning the immune response by the epithelial cells against infection and the roles of miRs in normal lung development and asthma [25]. It has been claimed that the miRs are critical for lung development and for maintaining disease-free lungs as shown by comparisons of normal lung tissue and tissue from asthmatic patients that revealed significant differences in miR profiles and suggest that miRs serve as a regulatory layer in the pathogenesis of asthma [25].

Also, different research studies demonstrated that in between the in vitro infection of primary airway epithelial cells from asthmatics and healthy adults with HRV, asthmatic cells produce fewer IFN-β and IFN-λ. It makes them potentially more susceptible to virus-induced cell cytotoxicity. IFN-β deficiency at asthma exacerbation promotes mixed lineage kinase domain-like protein (MLKL)-mediated necroptosis. Defective production of antiviral IFN-β is thought to contribute to rhinovirus-induced asthma exacerbations. Studies have shown that deficiencies in the IFN-α response of peripheral blood mononuclear cells and plasmacytoid dendritic cells are observed in asthmatic adults and children in response to RSV, HRV, and influenza A inoculation [25].

Moreover, the relationship between reduced FEV1 and exacerbation risk promotes long-term reductions in lung function. Alternatively, there may be a result of specific types of airway inflammation or host factors, like smoking, that result in a decline in lung function. Many experiments have provided evidence that factors such as allergy and baseline FEV1 can influence the changes in the lower-airway physiology caused by rhinovirus infection and may contribute to the increased lower-airway effects of rhinovirus infection in subjects with asthma [28].

In addition, cytokines, such as IL-11 level, in nasal secretions, were significantly increased in patients who demonstrated wheezing [28]. NF-κB activation appears to be essential for the rhinovirus-induced synthesis of proinflammatory cytokines such as IL-6 and IL-8 [28]. On the other hand, depleted IgG levels were noted in patients suffering from asthma exacerbations with virus-positive samples. However, there was no relationship with viral load, IFN-α, IFN-γ, IL-5, or IL-13 levels [21,22]. Moreover, in patients with severe asthma, deficiency of TLR7 in macrophages deteriorates innate immunity [27].

All in all, changes in innate immunity, IFN deficiencies, and altered immunoglobulins levels along with disrupted airway barriers make asthmatics increasingly prone to viral upper respiratory infections.

Limitations

The findings of this review should be considered in light of its limitations. The inclusion criteria for this review are very selective, and unpublished articles and articles in languages other than English have been excluded, increasing the probability of publication bias. The sample sizes of the studies screened in this review vary widely and affect the reliability of the results to a certain degree. Many more studies are needed to be conducted in the future on the asthmatic population, to produce more precise results applicable to a broader population in this regard.

## Conclusions

Based on the results of this review, we conclusively say that asthmatic patients are at a higher risk of contracting viral infections. The various factors contributing to the susceptibility in the asthmatic population are altered immune responses, decreased circulating antibodies, and the disruption of the physiological airway barrier. The viral strains that are commonly found as causative agents in patients with asthma include HRV, RSV, coronavirus, and enterovirus.

This represents an additional cause to enhance the demonstrated higher prevalence of exacerbations on asthmatics produced by the respiratory virus and points out a vicious-cycle theory of viral infection-asthma-viral infection.
